# Apps With Maps—Anxiety and Depression Mobile Apps With Evidence-Based Frameworks: Systematic Search of Major App Stores

**DOI:** 10.2196/16525

**Published:** 2020-06-24

**Authors:** Jamie M Marshall, Debra A Dunstan, Warren Bartik

**Affiliations:** 1 School of Psychology Faculty of Medicine and Health University of New England Armidale Australia

**Keywords:** mHealth, apps, app store, depression, anxiety, e-mental health, smartphone, mobile mental health, digital mental health, mobile phone

## Abstract

**Background:**

Mobile mental health apps have become ubiquitous tools to assist people in managing symptoms of anxiety and depression. However, due to the lack of research and expert input that has accompanied the development of most apps, concerns have been raised by clinicians, researchers, and government authorities about their efficacy.

**Objective:**

This review aimed to estimate the proportion of mental health apps offering comprehensive therapeutic treatments for anxiety and/or depression available in the app stores that have been developed using evidence-based frameworks. It also aimed to estimate the proportions of specific frameworks being used in an effort to understand which frameworks are having the most influence on app developers in this area.

**Methods:**

A systematic review of the Apple App Store and Google Play store was performed to identify apps offering comprehensive therapeutic interventions that targeted anxiety and/or depression. The PRISMA (Preferred Reporting Items for Systematic Reviews and Meta-Analyses) checklist was adapted to guide this approach.

**Results:**

Of the 293 apps shortlisted as offering a therapeutic treatment for anxiety and/or depression, 162 (55.3%) mentioned an evidence-based framework in their app store descriptions. Of the 293 apps, 88 (30.0%) claimed to use cognitive behavioral therapy techniques, 46 (15.7%) claimed to use mindfulness, 27 (9.2%) claimed to use positive psychology, 10 (3.4%) claimed to use dialectical behavior therapy, 5 (1.7%) claimed to use acceptance and commitment therapy, and 20 (6.8%) claimed to use other techniques. Of the 162 apps that claimed to use a theoretical framework, only 10 (6.2%) had published evidence for their efficacy.

**Conclusions:**

The current proportion of apps developed using evidence-based frameworks is unacceptably low, and those without tested frameworks may be ineffective, or worse, pose a risk of harm to users. Future research should establish what other factors work in conjunction with evidence-based frameworks to produce efficacious mental health apps.

## Introduction

### Background

The practice of psychotherapy is underpinned by evidence-based therapies and interventions. These techniques are used when they are proven to be efficacious via thorough experimental methods. In most developed countries, mental health clinicians are discouraged by professional associations and government regulations from using therapies without such evidence.

New technology is changing the way therapy can be delivered. Therapies previously only delivered face-to-face can now be accessed electronically, and the way that many people do this is via their smartphone. Smartphone apps are software programs that can be downloaded from specialist websites, known as app stores. The two biggest app stores are the Apple App Store and Google Play. Global expenditure on apps in 2018 was approximately US $92.1 billion [1], which attests to the widespread use of apps and value of smartphones to consumers.

### Mental Health: Is There an App for That?

Health, including mental health, apps are emerging as one of the most important categories of apps. A total of 75% of consumers believe that technology is important in managing their health, and 88% are willing to share mobile health (mHealth) data with their health care provider [2]. Approximately 48% of consumers are currently using a health app [2], and many more have downloaded at least one [3]. Health apps are in the top-10 list of worldwide categories for consumer spending on apps [4]. Although definitions about what constitutes a *health* app varies, and this influences the estimates of how many health apps are available, a 2017 report calculated that there were approximately 318,000 health apps available for download [5], with 10,000 of these relating to mental health [6].

People with mental illness are attracted to the possibility of utilizing apps to manage their mental health [7]; however, there is doubt in this population about how apps manage confidential information [8]. There is also uncertainty about the available functions of mental health apps and which apps are the most useful [9]. For guidance about what app to download, mental health consumers rely mainly on ratings and reviews in app stores [10] or on advice given through social media or word of mouth [11]. However, if a mental health expert is not involved in the development of an app, and if it has not been developed using an evidence-based theoretical framework, the app may not be effective or, worse, may do harm to its user.

There are many reasons why research on the efficacy of apps for anxiety and/or depression should be prioritized. Firstly, many people are already using mental health apps [6], and with five billion people around the world currently using a smartphone [12], there is potential for many more to do so in the future as this figure rises. Apps provide greater accessibility to mental health resources and offer instant mental health assistance. These features are especially relevant for traditionally difficult-to-reach cohorts, such as teenagers [13] and people in rural communities [14]. Mental health apps may also offer more cost-effective options for lower socioeconomic groups [15] and greater anonymity and flexibility for others. For example, using an app may be more convenient for consumers with limited time to access other therapy, and more convenient for clinicians who may prefer their clients and patients to do homework activities on their smartphone so that results can be digitally sent back to the clinician. Certain clients in therapy may also just prefer the novel value of doing interactive homework activities on their phone, rather than with a pen and paper. For many people, being able to use their phone for such activities is simply more convenient (eg, it may be easier for them to use a smartphone to do homework while they are travelling on a bus rather than handling awkward pieces of paper in such situations). In this way, apps may be used as adjuncts to other therapies. Apps can also be used in more practical ways, such as setting reminders to assist with treatment and medication compliance. All of these factors create the potential for apps to reduce the burden on existing mental health services [16].

### Apps and Evidence-Based Frameworks

For a mental health app to be efficacious, it is fundamental that it be underpinned by an evidence-based framework; that is, an established therapy technique for reducing psychological distress, such as cognitive behavioral therapy (CBT). An evidence-based framework provides a road map or blueprint for an app’s functions and performance. However, even when an app claims to offer CBT, the contents and functioning of the app may not align with CBT principles [17], especially if the app has been developed without expert input.

To date, there has been little research that addresses the potential harm a mental health app may do to an individual. It seems conceivable, however, that an app developed without a proven theoretical framework and method of intervention could have the potential to do harm to a user [18]. For instance, a recommendation to use herbal supplements could adversely interact with prescribed medication.

Over the last two decades, e-mental health programs with a CBT framework and designed for use on computers have been found to be effective for both adults and children in reducing anxiety and depression [19,20] and improving happiness and well-being [21-23]. Some studies have compared the effectiveness of e-mental health programs with face-to-face therapy and have found comparable [24,25], and, in some cases, even more favorable, results [26]. There is now an interest in how stand-alone apps for smartphones (ie, programs that can be downloaded and used without an internet connection) based on CBT or other evidence-based theoretical frameworks may further enhance a clinician’s digital toolbox [27].

CBT proposes that emotions can be more effectively managed by adjusting thinking and behavior and recognizing physiological responses [28]. CBT has an established evidence base and history of successfully treating anxiety and depression, both in individual therapy [29] and in group settings [30]. The CBT framework offers a broad range of efficacious interventions [31], including the following: the use of *thought diaries* to challenge negative thinking; coming up with personally meaningful affirmations; different types of physical exercise, such as walking and dancing; increasing social connectedness and having personally meaningful social interaction; increasing the amount of time doing pleasurable, or once pleasurable, activities; structured problem solving; and others. Many of these CBT interventions have been incorporated into mental health apps.

Other therapies that have been developed from traditional CBT have also been used successfully to treat anxiety and depression. Positive psychology, for instance, has become a more recent framework that is, under a strict definition, a type of CBT but has a different focus. Rather than simply *fixing* psychopathology, positive psychology aims to help individuals reach their full well-being potential by increasing optimism and happiness [32]. Studies on various positive psychology interventions have revealed efficacious empirical support for reducing symptoms of anxiety and depression as well as increasing well-being and happiness. For example, Freedman and Enright [33] showed that forgiveness significantly decreased depression, Froh et al [34] implemented a gratitude intervention that resulted in significantly greater optimism and life satisfaction as well as decreased negative affect, Lyubomirsky et al [35] reported an increase in happiness and well-being for participants who completed acts of kindness, and Seligman et al [36] successfully used three strategies to increase happiness and decrease depressive symptoms: (1) using one’s signature strengths in new ways, (2) writing down three positive things each day, and (3) writing a letter of gratitude. All of these interventions could conceivably be incorporated into mental health apps and most already have.

Similarly, other frameworks, often referred to as third-wave therapies [37], have offered up interventions that are suitable for use in mental health apps. Various mindfulness interventions have proved efficacious in the past [38], and these meditation and breathing activities are easily incorporated into an app. Dialectical behavior therapy (DBT) is an intersection of various interventions, including mindfulness, distress tolerance, emotional regulation, and improving social relatedness activities. These may involve dialectical interaction with a therapist, including recording distress ratings, among other interventions that have proven effective in reducing symptoms of anxiety and/or depression, with particular focus on individuals with a diagnosis of borderline personality disorder [39]. Interpersonal therapy (IPT) involves interventions designed to improve current interpersonal relationships and social dysfunction, rather than focusing on personality, as an efficacious way of improving symptoms of depression [40]. Acceptance and commitment therapy (ACT) focuses on an individual accepting distressing thoughts and performing committed actions guided by one’s values; ACT has proven efficacious for both anxiety [41] and depression [42]. All the frameworks listed here potentially comprise interventions that may conceivably be incorporated into an app.

### Objectives of This Study

This study involved a systematic search of app stores and concentrated on apps that offered a comprehensive therapeutic treatment for anxiety and/or depression, as opposed to apps that may offer singular or novel interventions (see *inclusion criteria* in the Methods section for more details about this definition). The research questions were as follows: (1) What proportion of publicly available apps offering a therapeutic treatment for anxiety and/or depression have used an evidence-based framework in their development? and (2) In an effort to understand which specific frameworks are having the most influence on mental health app developers, what are the proportions of specific frameworks? No previous study or review could be located that examined this issue by focusing on publicly available apps listed in the app stores.

## Methods

This systematic review used the AMSTAR (A MeaSurement Tool to Assess systematic Reviews) 2 [43] and PRISMA (Preferred Reporting Items for Systematic Reviews and Meta-Analyses) [44] protocols for guidance in conducting the app store search. Although these protocols were developed specifically for literature searches, they offer appropriate direction that can be applied to searching app stores in the absence of such a specific guide. However, there are limitations in their use for guidance in app store searches. We adapted both of these protocols into a unique protocol and checklist specifically for app store searches: the Protocol for App Store Systematic Reviews (PASSR) checklist is a combination and reworking of the items from AMSTAR 2 and PRISMA that can be applied to the systematic search of app stores for any category of apps. The same wording as used in both AMSTAR 2 and PRISMA has been retained wherever possible to enable ease of comparison (see Multimedia Appendix 1).

Four researchers, including the lead author (JMM), systematically searched the Apple App Store and Google Play store in December 2018, and then again in July 2019, to ensure newly available apps were included for review. These two marketplaces attract more than three times as much revenue from downloads as their next competitor, the Windows Store [45], and over 90% of available apps for depression can be found in either of these stores [46].

Searches in each marketplace were made with the following 19 keywords across all categories: mental health, depression, anxiety, wellbeing, happiness, psychological distress, positive psychology, suicide, mental illness, CBT, cognitive behaviour therapy, cognitive behavior therapy, ACT, acceptance and commitment therapy, DBT, dialectical behaviour therapy, dialectical behavior therapy, IPT, and interpersonal therapy.

Apps were shortlisted based on their app store descriptions, and apps that were available in both stores were only counted once.

Inclusion criteria were as follows:

1. The app language and store description are in English.
2. The app offers a therapeutic treatment for anxiety and/or depression, not just singular elements of a therapy, such as monitoring symptoms, recording thoughts, or diagnosing the disorder, although apps could have any of these as part of a therapeutic treatment. In this way, apps were excluded if they used only singular elements. This approach is in contrast to other similar reviews [27] that have identified apps with single tools rather than comprehensive treatments. Therapeutic treatment was defined for this study’s purpose as “offering focused treatment suggestions specifically for reducing symptoms of anxiety and/or depression in a manner comprehensive enough to be considered a type of therapy.”

## Results

A search of the Apple App Store and Google Play store uncovered a shortlist of 293 apps whose app store descriptions inferred that they offered a therapeutic treatment for anxiety and/or depression; the full list of these apps is available from the corresponding author (JMM). Of these 293 apps, a total of 162 (55.3%) claimed to have an evidence-based theoretical framework informing the app’s development. Differences between the Apple App Store (112/197, 56.9%) and the Google Play store (50/96, 52%) were negligible.

The evidence-based frameworks found in the apps represented the following proportions: CBT, 30.0% (88/293); mindfulness, 15.7% (46/293); positive psychology, 9.2% (27/293); DBT, 3.4% (10/293); ACT, 1.7% (5/293); and others, 6.8% (20/293). Note, some apps claimed to use multiple frameworks—each time a framework was mentioned, it was counted.

When including only the 162 apps with a theoretical framework in the analysis, the breakdown was as follows: CBT, 54.3% (88/162); mindfulness, 28.4% (46/162); positive psychology, 16.7% (27/162); DBT, 6.2% (10/162); ACT, 3.1% (5/162); and others, 12.3% (20/162).

Of those 162 apps with evidence-based frameworks, 10 (6.2%) were found to have published evidence for their effectiveness (see Table 1). The nine research articles [47-55] that examined the 10 apps were made up of seven randomized controlled trials (RCTs)—the Flett et al article contained one RCT with 2 of the listed apps: *Headspace* and *Smiling Mind* [52]—and two feasibility or pilot studies: Kinderman et al [48] and Carey et al [55]. Total participant numbers were comprised of 1017 in intervention conditions and 447 in control groups. The mean age across those studies that provided enough information was 32.7 years (SD 9.3). Intervention phases fluctuated between 2 and 12 weeks. Three studies reported long-term follow-up, the longest period being 3 months (see Table 2 for a summary of research characteristics).

**Table 1:** Coding used for app store search, and the search results.

Code	Description (ie, framework)	Apps with this framework (N=293), n (%)	Apps with published research, n (%)	Name of the app (or apps) with published research
CBT	Cognitive behavioral therapy	88 (30.0)	5/88 (6)	Agoraphobia Free Catch It PTSD Coach MoodMission Thought Challenger
MIND	Mindfulness	46 (15.7)	3/46 (7)	Headspace Smiling Mind DeStressify
POS	Positive psychology	27 (9.2)	1/27 (4)	SuperBetter
DBT	Dialectical behavior therapy	10 (3.4)	0 (0)	N/A^a^
ACT	Acceptance and commitment therapy	5 (1.7)	0 (0)	N/A
OTH	Other recognized framework	20 (6.8)	1/20 (5)	MindSurf
NONE	No theoretical framework	131 (44.7)	0 (0)	N/A

^a^N/A: not applicable.

**Table 2:** Summary of published research for shortlisted apps.

App name	Reference	Sample characteristics	Intervention period; long-term follow-up period	Statistically significant improvements	Outcome measure used
Agoraphobia Free	Christoforou et al [47]	Intervention group: n=73 Control group: n=69 Mean age (SD): 39.7 years (11.3)	12 weeks; no follow-up	Anxiety	PAS^a^
Catch It	Kinderman et al [48]	Intervention group: n=285 Control group: none Mean age (SD): 48.2 years (SD not given)	6 weeks (but varied among participants); no follow-up	*Positive* and *negative* mood	None^b^
PTSD Coach	Kuhn et al [49]	Intervention group: n=62 Control group: n=58 Mean age (SD): 39.3 years (14.6)	3 months; 3 months	Anxiety and depression	PCL-C^c^ and PHQ-9^d^
MoodMission	Bakker et al [50]	Intervention group (a): n=56 Intervention group (b): n=56 Intervention group (c): n=50 Control group: n=64 Mean age (SD): 34.2 years (12.1)	30 days; no follow-up	Depression	PHQ-9
Thought Challenger	Stiles-Shields et al [51]	Intervention group (a): n=10 Intervention group (b): n=10 Control group: n=10 Mean age (SD): not given	6 weeks; 4 weeks	Depression	PHQ-9
Headspace	Flett et al [52]	Intervention group: n=67 Control group: n=67 Mean age (SD): 20.1 years (2.8)	40 days; no follow-up	Depression	CES-D^e^
Smiling Mind	Flett et al [52]	Intervention group: n=58 Control group: n=67 Mean age (SD): 20.1 years (2.8)	40 days; no follow-up	Depression	CES-D
DeStressify	Lee and Jung [53]	Intervention group: n=77 Control group: n=86 Mean age (SD): 20.6 years (SD not given)	4 weeks; no follow-up	Trait anxiety	STAI^f^
SuperBetter	Roepke et al [54]	Intervention group (a): n=93 Intervention group (b): n=97 Control group: n=93 Mean age (SD): 40.2 years (12.4)	4 weeks; 6 weeks	Depression	CES-D
MindSurf	Carey et al [55]	Intervention group: n=23 Control group: none Mean age (SD): not given	2 weeks; no follow-up	Anxiety and depression (not statistically significant)	DASS-21^g^

^a^PAS: Panic and Agoraphobia Scale.

^b^Used direct data input from apps based on words used by users to describe mood.

^c^PCL-C: PTSD (posttraumatic stress disorder) Checklist – Civilian.

^d^PHQ-9: Patient Health Questionnaire – Depression Scale.

^e^CES-D: Center for Epidemiologic Studies – Depression Scale.

^f^STAI: State Trait Anxiety Inventory.

^g^DASS-21: Depression Anxiety Stress Scales – 21-Item Version.

## Discussion

### Principal Findings

The purpose of this study was to locate mobile mental health apps for treating anxiety and/or depression that contained recognized theoretical frameworks underpinning their development. A search of the Apple App Store and Google Play store revealed a total of 293 apps claiming to offer a therapeutic treatment for anxiety and/or depression, with just over half claiming to have been developed using an evidence-based theoretical framework. Of these, CBT was the most quoted framework in app store descriptions. The method used to identify these apps mimicked that of how a consumer in the general population would ordinarily locate an app to treat anxiety and/or depression; that is, by using the search function of an app store, then reading the description of each app.

CBT has a research history of over 60 years, longer than any of the other theoretical frameworks contained in the shortlisted apps [56]. It is widely known as being effective for a range of conditions, including cessation of smoking [57] and other drugs [58], managing pain [59], and relieving symptoms of mental ill-health across a range of psychological disorders [56]. CBT is also a term widely known across health-related settings and professions and is often used in information directed at the general community for public campaigns of ways to manage mental ill-health and stress [60]. It perhaps comes as no surprise, then, that CBT is the most widely used theoretical framework in apps for anxiety and depression. However, new research is accelerating in many of the other theoretical frameworks listed in this review [61]; it may be that the proportion of CBT-based apps lessens over time as a result.

The popularity of the term CBT, as well as the growing popularity of the terms used to identify the other evidence-based frameworks in this review, may be contributing to apps being developed by nonexperts who incorrectly quote these terms in app store descriptions as a means of attempting to gain legitimacy. There are no known safeguards in place anywhere in the world to stop this from happening. While government agencies have started to regulate the health app space, this regulation has thus far focused on apps that only pose a risk of harm to users [62]. While this is important and a welcome addition to the oversight of mental health apps, it does not provide checks on accuracy of information in app store descriptions of apps that may not fall into the category of posing a risk of harm. Such apps may provide fake or incorrect information that may, at worst, be ineffective at reducing anxiety or depression, but they may continue to be available in app marketplaces because they do not meet criteria that would identify them as posing a risk of harm to users. If an app claims to wrongly categorize its interventions as CBT, DBT, ACT, mindfulness, or positive psychology, one of the dangers is that such misuse of these terms may lead users to believe that such theoretical frameworks do not work if the user does not get any benefit from the app.

A detailed analysis of the research that accompanied 10 of the shortlisted apps is outside the scope of this paper, as this review is focused on the theoretical frameworks that underpin mental health apps. The quality and quantity of research into mental health apps has been detailed elsewhere [63-66]. However, the research found to accompany the apps listed here does appear to vary greatly in methodology; this reaffirms the claims of heterogeneity made in those literature reviews. These reviews all call for more research and ongoing evaluation of research methodology into studying apps, as the current methodologies may not be the most appropriate [62,67,68].

By examining evidence-based frameworks in mental health apps, this review has highlighted the high proportion (131/293, 44.7%) of apps that claim to offer a therapeutic treatment for anxiety and/or depression that do not rely on validated techniques. It is useful to think of an evidence-based theoretical framework as being like a map that guides clinicians in their therapeutic practices. This is at the heart of a clinician being effective in their treatment and, at the very least, not doing harm to their client or patient. While many mental health apps claim to be using an evidence-based theoretical framework, as many as 44.7% may not be; this leaves open the possibility, therefore, that a large proportion of these apps may be ineffective and possibly run the risk of doing the user harm.

### Limitations

This review of app stores suffers from the same limitation as other reviews of app stores: the restricted way that searches are conducted. The Apple App Store and Google Play store search results can be challenging because important information may be absent. How developers have completed online questionnaires prior to registering their app for public download, as well as the interaction of these with algorithms developed by the app store, determine the outcomes of a search. Consequently, there are differences in the order in which apps are presented when specific search terms are employed, and there are limited search functions compared to those available when performing a literature search. The results of an app store search are not necessarily presented in a logical order to users because they are unable to choose to display results according to multiple criteria, such as being able to filter from most recent to oldest, as one can do in a literature search. The outcome can lead to considerable ambiguity about the order of displayed results.

Another difference between a search of app stores and a search of the literature is that research may be found on a particular app, but that app may not be available for download to the general public. For example, Torous et al [27] discovered peer-reviewed publications on the efficacy of four CBT-based apps—excluding apps that were based on DBT or ACT—but when they searched the app stores for these apps, none could be found. While research is welcomed and encouraged in the development of mental health apps, in the end what matters most is how many of these efficacious apps are available to the general population.

Another limitation of this research is that results were based on the contents of descriptions in the app stores. None of the shortlisted apps were trialed to confirm that their description corresponded to actual content. For example, a nonexpert may have developed an app and claimed in the description that it used a CBT framework, but the developer may not have incorporated any genuine CBT interventions into the app functions. If such pseudo-CBT apps exist, it is likely that they will fail to assist users to manage anxiety and/or depression and may, therefore, lead consumers to believe that CBT is ineffective [27].

### Conclusions

This review has highlighted difficulties faced by clinicians and consumers when searching the app stores for an app that offers a therapeutic treatment for anxiety and/or depression. The limited search capabilities of the app stores make it difficult to find the most appropriate app for one’s needs. If an individual wants to find a mental health app based on an evidence-based framework, it is difficult to sort through the many other apps that do not have that guiding framework.

Just as mental health clinicians are trained to follow evidence-based frameworks in their practice, it is reasonable to assume that mental health apps should do the same in their functioning. This review found that little more than half do, according to their app store descriptions. Just as successful therapeutic outcomes of face-to-face therapy can be attributed to more than the theoretical framework—factors such as rapport with the therapist, therapist skills, and an individual’s motivation to change—so too are there other elements of a mental health app that may contribute to a successful therapeutic outcome, such as usability and ease of use, aesthetics, level of gamification, etc [65,69]. There would appear to be much research that still needs to be done on all these factors and how they interrelate with theoretical frameworks as well as whether certain factors mediate or moderate others.

Another suggested area of future research is to compare apps developed with evidence-based theoretical frameworks with their face-to-face equivalents. Clinicians and consumers need to know about the effectiveness and limitations of apps and where they sit alongside traditional evidence-based approaches. If clinicians and consumers become more confident in understanding how mental health apps can assist in reducing symptoms of anxiety and/or depression, it may increase the take-up of this new treatment modality and turn the potential advantages of using mental health apps into a reality.

## Appendix

Multimedia Appendix 1 Protocol for App Store Systematic Reviews (PASSR).

## Abbreviations

ACT: acceptance and commitment therapy
AMSTAR: A MeaSurement Tool to Assess systematic Reviews
CBT: cognitive behavioral therapy
DBT: dialectical behavior therapy
IPT: interpersonal therapy
mHealth: mobile health
PASSR: Protocol for App Store Systematic Reviews
PRISMA: Preferred Reporting Items for Systematic Reviews and Meta-Analyses
RCT: randomized controlled trial
